# Silent Colistin Resistance by VITEK 2 Compact Versus Broth Microdilution in Bloodstream *Klebsiella pneumoniae* and *Escherichia coli*: A Prospective Single-Centre Study

**DOI:** 10.3390/antibiotics15101019

**Published:** 2026-10-14

**Authors:** Yasemin Ay Altıntop, Ekin Kırbaş, Banu Sancak

**Affiliations:** 1 Department of Medical Microbiology, Kayseri City Hospital, Kayseri Medical School, University of Health Sciences, 38080 Kayseri, Türkiye; 2 Department of Medical Microbiology, Faculty of Medicine, University of Eskişehir Osmangazi, 26040 Eskişehir, Türkiye; 3 Department of Medical Microbiology, Faculty of Medicine, Hacettepe University, 06100 Ankara, Türkiye

**Keywords:** colistin resistance, *Klebsiella pneumoniae*, bloodstream infection, bacteremia, broth microdilution, VITEK 2, antimicrobial susceptibility testing, very major error, antimicrobial stewardship, antimicrobial resistance surveillance

## Abstract

**Background/Objectives:** Colistin remains a last-resort therapy for carbapenem-resistant *Klebsiella pneumoniae* bloodstream infections, yet its accurate reporting depends on the reference broth microdilution (BMD) method that few clinical laboratories routinely deploy. We aimed to determine the prevalence of colistin resistance in bloodstream isolates and to quantify the diagnostic performance of the widely used VITEK 2 Compact automated system in a Turkish tertiary-care hospital. **Methods:** We prospectively analysed 253 consecutive non-duplicate blood culture isolates (144 *K. pneumoniae*; 109 *Escherichia coli*) obtained at a Turkish tertiary-care hospital between July 2022 and March 2023. Paired colistin susceptibility testing used BMD (EUCAST guidance, polypropylene plates, no surfactant) and VITEK 2 Compact with AST-N326 cards. EUCAST v12.0 breakpoints (susceptible ≤ 2 mg/L; resistant > 2 mg/L) were applied. Very major error (vME) rate was benchmarked against the ISO 20776-2 acceptance threshold of 3%. **Results:** BMD identified colistin resistance in 22.5% (95% CI 17.8–28.1) of isolates, with a striking inter-species disparity: 38.2% for *K. pneumoniae* versus 1.8% for *E. coli* (Fisher exact *p* = 1.1 × 10^−13^; OR 33.1, 95% CI 8.0–138). VITEK 2 misclassified 32 of 57 truly resistant isolates as susceptible; under the ISO 20776-2 denominator the very major error rate was 56.1% (32/57; 95% CI 43.3–68.3), approximately 18-fold above the ISO acceptance threshold of 3% (*p* < 0.0001). Overall sensitivity for colistin resistance detection was 43.9% (25/57); for *K. pneumoniae* alone, sensitivity was 45.5% (25/55) and specificity 95.5% (85/89), and every skipped-well BMD isolate (*n* = 11) was reported susceptible by VITEK 2. **Conclusions:** Automated colistin testing underdetects resistant *K. pneumoniae* bloodstream isolates in a high-prevalence setting. Our findings support continued adherence to BMD as the reference standard for colistin susceptibility in such settings, with automated systems used for screening and complemented by reference-method confirmation of critical isolates.

## 1. Introduction

Antimicrobial resistance (AMR) is now a leading global health threat, responsible for an estimated 4.95 million deaths in 2019 and 1.27 million deaths directly attributable to resistant bacterial infections [1].

Within this global picture, carbapenem-resistant Enterobacterales (CRE) have emerged as a critical priority pathogen, and the World Health Organization has included carbapenem-resistant *Klebsiella pneumoniae* in the highest-priority tier of its 2024 bacterial priority pathogens list requiring urgent research and development of new antimicrobial agents [2]. Bloodstream infections caused by carbapenem-resistant *K. pneumoniae* carry pooled mortality exceeding 40%, with intensive-care, haemato-oncology, and post-surgical populations disproportionately affected [3].

Although modern treatment guidelines increasingly favour newer β-lactam/β-lactamase inhibitor combinations for CRE infections, many middle-income settings, including Türkiye and neighbouring countries in southeastern Europe, the Balkans, and the Middle East, still rely on colistin as the last available option for definitive therapy [4,5]. Under these conditions, accurate colistin susceptibility reporting is not merely a laboratory concern but a clinical necessity that directly determines patient outcomes, and it is also a public-health necessity that determines the accuracy of regional AMR surveillance data used to shape empirical treatment policy.

Colistin susceptibility testing remains one of the most technically demanding procedures in the clinical microbiology laboratory. The amphipathic structure of the molecule causes it to bind polystyrene surfaces and gradient strips, generating artefactually low minimum inhibitory concentration (MIC) values. Agar dilution, disc diffusion, and gradient diffusion methods have therefore been formally deprecated, and rapid tests developed for polymyxin detection have offered only partial resolution [6]. Broth microdilution (BMD) performed in polypropylene microplates without surfactants is currently the sole reference method endorsed by both EUCAST and CLSI [7]. Despite this recommendation, routine BMD adoption remains low, and the majority of clinical laboratories in the region depend on automated platforms, predominantly the VITEK 2 Compact system (bioMérieux), for daily colistin reporting [8].

Heteroresistance, in which a phenotypically susceptible bacterial population harbours subpopulations capable of growth at elevated colistin concentrations, adds a further analytical dimension. On BMD reading, such isolates may show a skipped-well pattern, with growth in a higher concentration well appearing beyond one or more inhibited wells. This pattern is suggestive of, rather than diagnostic for, heteroresistance; formal confirmation requires population analysis profiling. Its clinical impact is well documented: colistin monotherapy in such cases often selects for the resistant subpopulation, leading to on-therapy failure and eventual emergence of frank resistance [9,10].

Turkish national antimicrobial resistance surveillance data indicate a steady rise in colistin resistance rates for *K. pneumoniae* over the past two decades, with a recent systematic review and meta-analysis reporting a pooled prevalence of approximately 1.6% but with marked heterogeneity and a clear temporal trend upward, particularly in tertiary-care and intensive-care settings [11]. Regional data from the WHO Global Antimicrobial Resistance and Use Surveillance System (GLASS) and the European Antimicrobial Resistance Surveillance Network (EARS-Net) indicate that colistin-resistant *K. pneumoniae* is emerging as a public-health concern across the Balkans, southeastern Europe, and the eastern Mediterranean [12,13]. However, the reliability of such surveillance data critically depends on the diagnostic accuracy of the underlying laboratory methods, an assumption that has received limited scrutiny in Turkish cohorts.

*E. coli* is the single most common cause of community-acquired and nosocomial Gram-negative bloodstream infection worldwide, and colistin resistance in *E. coli* remains uncommon in most European surveillance programmes but is reported at a non-zero and locally variable rate across the EARS-Net reporting network [13]. Although the clinical urgency of a colistin susceptibility report is greatest for carbapenem-resistant *K. pneumoniae*, routine laboratory testing is applied to both species, so the diagnostic performance of any automated system must be evaluated across both.

Against this background, three linked questions remain underexplored: what is the phenotypic prevalence of colistin resistance in bloodstream *K. pneumoniae* and *E. coli* isolates when tested by the reference method, how reliably does the widely deployed automated system identify this resistance, and what are the consequences of any diagnostic gap for individual patient care, institutional antimicrobial stewardship, and regional AMR surveillance. To address these questions, we prospectively analysed 253 consecutive blood culture isolates of *K. pneumoniae* and *Escherichia coli* collected at a Turkish tertiary-care hospital and compared paired BMD and VITEK 2 Compact susceptibility results, focusing on clinical significance, diagnostic pitfalls, stewardship implications, and the reliability of regional resistance surveillance.

## 2. Results

### 2.1. Isolate Characteristics

**Figure 1:**
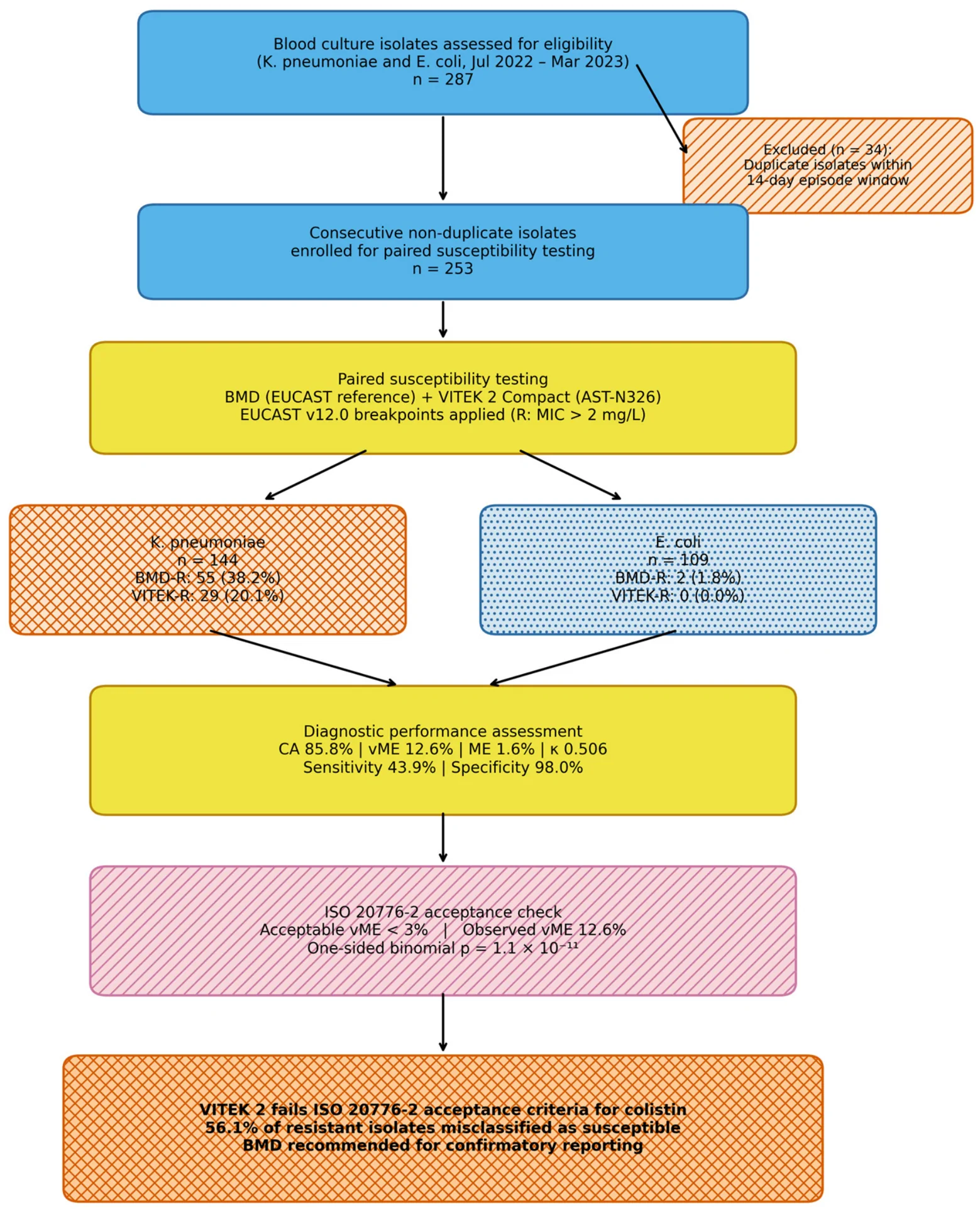
Study flow diagram in accordance with the STROBE guideline for observational studies. The 11 *K. pneumoniae* isolates with skipped-well patterns on BMD were retained in the primary analysis, with the highest observed MIC value assigned per protocol. In the diagram, filled boxes without hatching mark enrolment and testing steps, diagonal hatching marks exclusion and acceptance-check steps, cross-hatching marks the *K. pneumoniae* arm and the overall conclusion, and dotted shading marks the *E. coli* arm; these fill patterns are used in addition to colour to distinguish categories and remain legible for colour-blind readers. BMD, broth microdilution; CA, categorical agreement; ME, major error; vME, very major error.

From an initial pool of 287 *K. pneumoniae* and *E. coli* blood culture isolates identified during the study period, 253 consecutive non-duplicate isolates were included after exclusion of 34 duplicates. The final cohort comprised 144 *K. pneumoniae* (56.9%) and 109 *E. coli* (43.1%). Isolates originated from internal medicine (38.3%), general surgery (28.5%), intensive care (22.5%), and haematology-oncology (10.7%) services (Table 1). The full STROBE-compliant flow of isolate inclusion is shown in Figure 1. Paired BMD and VITEK 2 Compact susceptibility results were available for all 253 isolates, and no data were missing.

### 2.2. Substantial Colistin Resistance in Bloodstream K. pneumoniae

**Figure 2:**
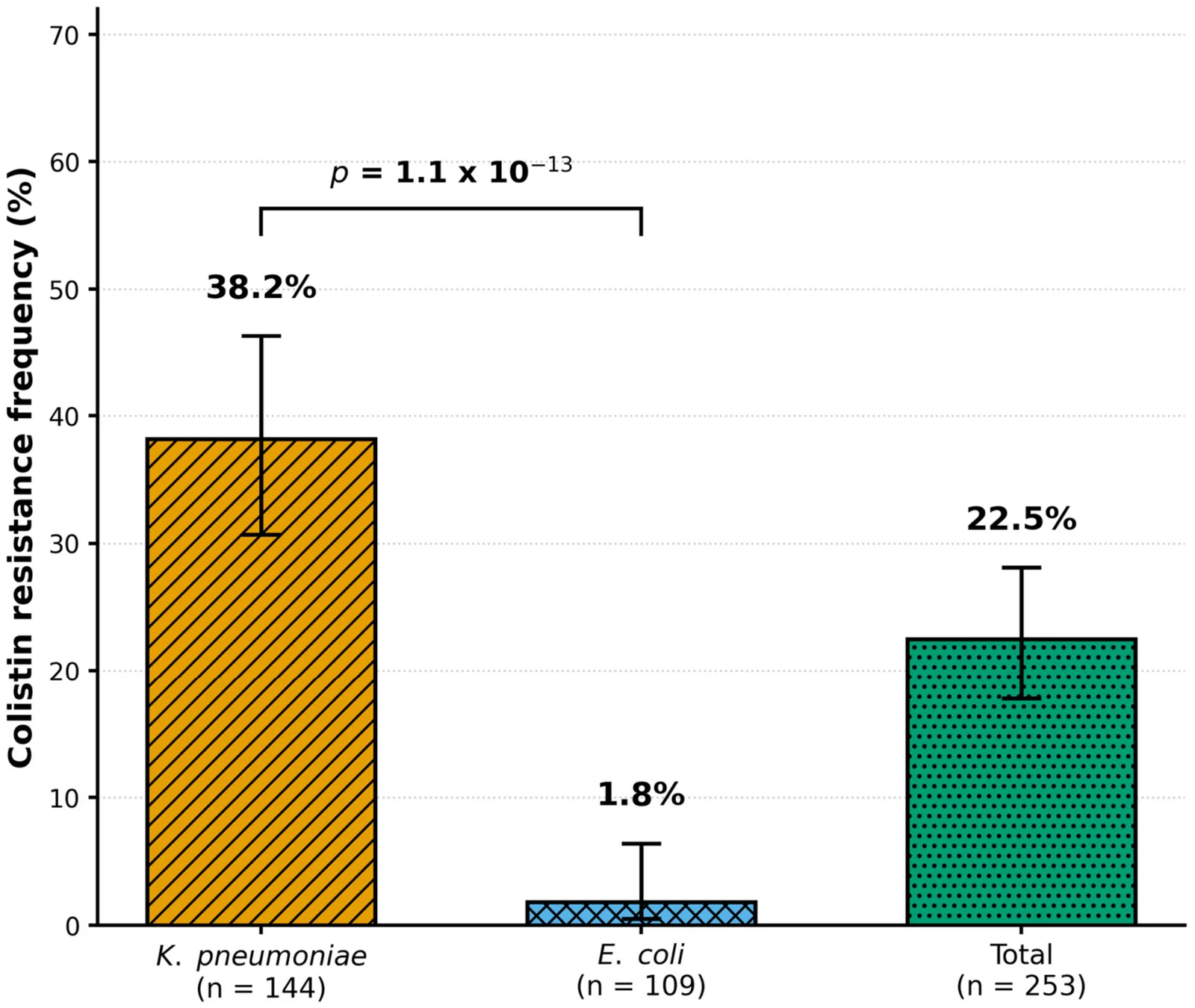
Colistin resistance frequency by species, determined by BMD (reference method). Error bars represent Wilson 95% confidence intervals. Resistance defined per EUCAST v12.0 breakpoints (MIC > 2 mg/L). Between-species comparison performed by Fisher exact test.

**Figure 3:**
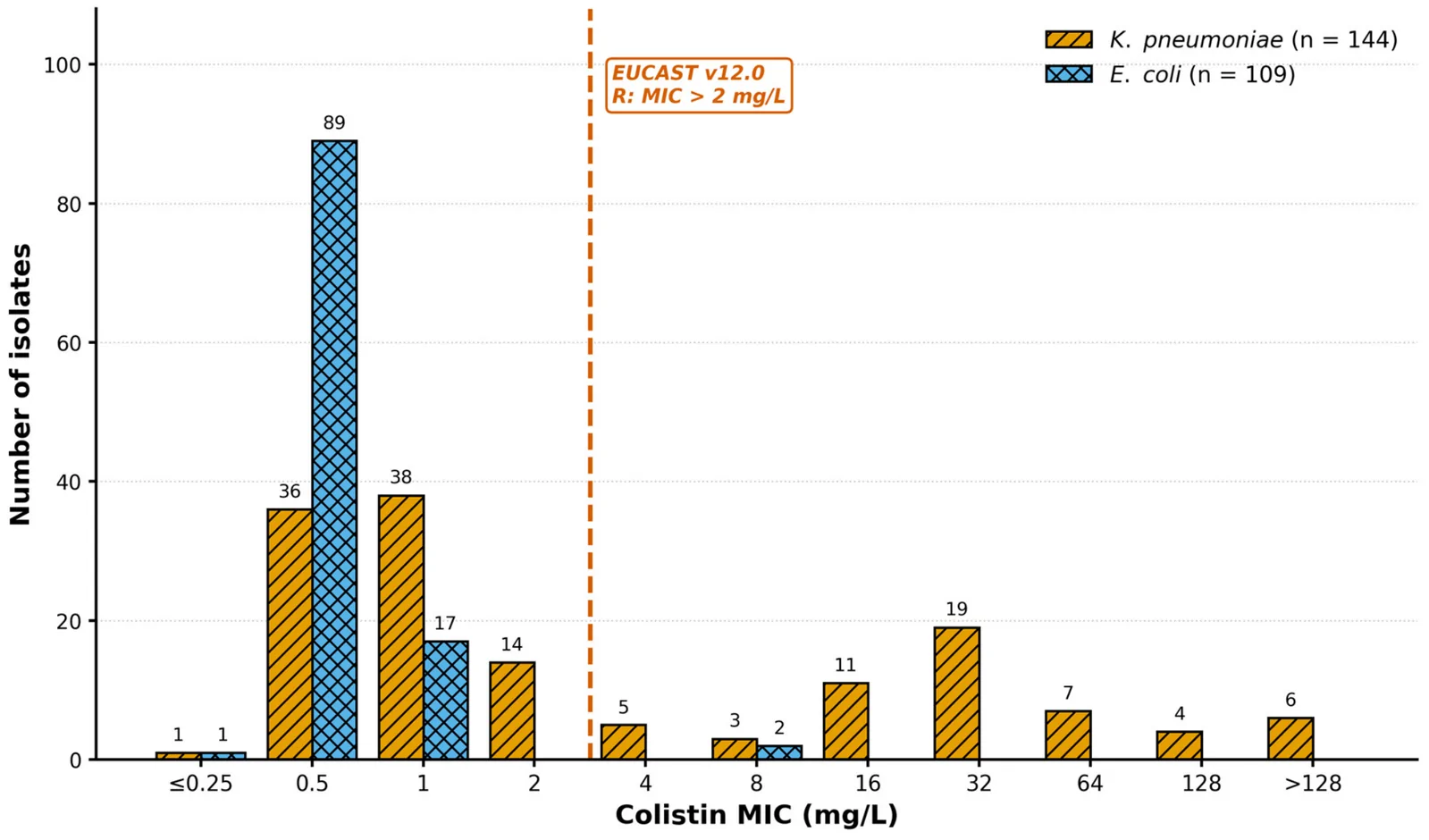
Distribution of colistin MIC values determined by BMD for *K. pneumoniae* and *E. coli* blood isolates (*n* = 253). The dashed vertical line indicates the EUCAST v12.0 resistance breakpoint (MIC > 2 mg/L). *K. pneumoniae* shows a broad, right-shifted distribution extending to >128 mg/L, whereas *E. coli* MICs are concentrated within the susceptible range.

BMD identified colistin resistance in 57 of 253 isolates, giving an overall phenotypic resistance frequency of 22.5% (95% CI 17.8–28.1). A striking inter-species disparity emerged, with 38.2% (55/144; 95% CI 30.7–46.3) of *K. pneumoniae* isolates classified as resistant compared with only 1.8% (2/109; 95% CI 0.5–6.4) of *E. coli* (Fisher exact *p* = 1.1 × 10^−13^; OR 33.1, 95% CI 8.0–138; Figure 2). The MIC distribution (Figure 3) demonstrated a broad, right-shifted profile for *K. pneumoniae* (MIC_50_ 1 mg/L, MIC_90_ 64 mg/L, range extending to >128 mg/L), whereas *E. coli* MICs were concentrated within the susceptible range (MIC_50_ 0.5 mg/L, MIC_90_ 1 mg/L). This 38% colistin resistance rate substantially exceeds the pooled national estimate of approximately 1.6% derived from a recent Turkish systematic review [11], suggesting the emergence of a new local high-prevalence phenotype that has direct implications for empirical therapy and regional AMR surveillance.

### 2.3. Diagnostic Underdetection by VITEK 2 Compact

**Figure 4:**
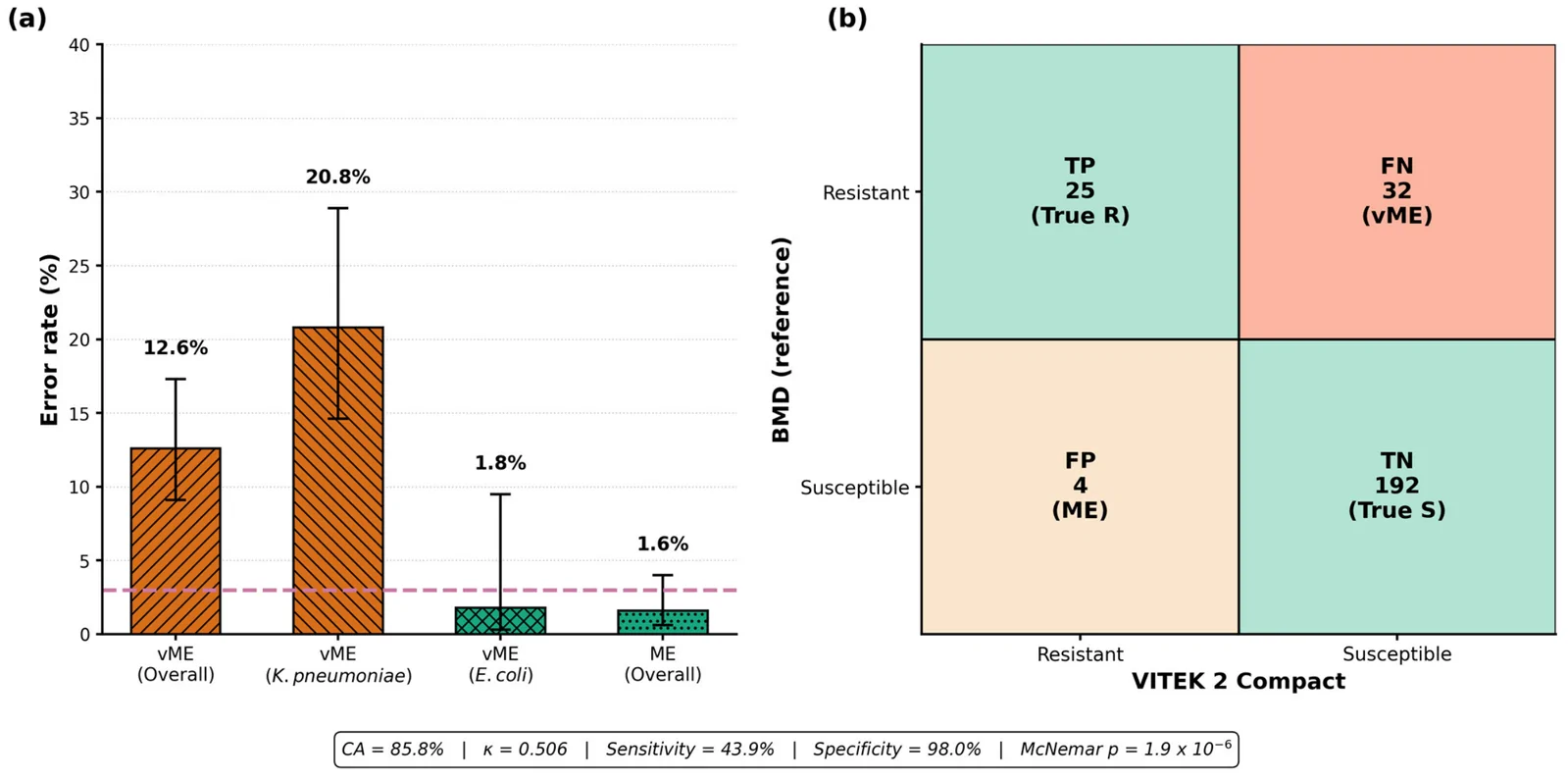
Diagnostic performance of VITEK 2 Compact against BMD (*n* = 253). (**a**) Very major error (vME) and major error (ME) rates, with Wilson 95% confidence intervals, benchmarked against the ISO 20776-2 acceptance threshold of 3% (dashed line). (**b**) Categorical agreement matrix showing that 32 of 57 truly resistant isolates were misclassified as susceptible by VITEK 2. Summary metrics are shown below the matrix.

The VITEK 2 Compact system classified only 29 of 253 isolates (11.5%, 95% CI 8.1–16.0) as resistant, capturing less than one third of the resistance burden identified by BMD. When disaggregated by species, VITEK 2 reported 29 *K. pneumoniae* (20.1%, 95% CI 14.4–27.4) and no *E. coli* (0.0%, 95% CI 0.0–3.4) as resistant, compared with BMD frequencies of 38.2% and 1.8%, respectively. Overall categorical agreement was 85.8% (217/253; 95% CI 80.9–89.5). Computed under the ISO 20776-2 denominator, the very major error rate reached 56.1% (32/57 resistant isolates; 95% CI 43.3–68.3), approximately 18-fold above the ISO acceptance threshold of 3% (one-sided binomial test, *p* < 0.0001). The major error rate remained within acceptable limits at 4.5% (4/89 susceptible *K. pneumoniae*; 95% CI 1.8–11.2). Cohen’s κ for method agreement was 0.506, indicating only moderate chance-corrected concordance. Exact McNemar testing confirmed highly significant systematic discordance (*p* = 1.9 × 10^−6^). Overall sensitivity for colistin resistance detection was 43.9% (25/57); for *K. pneumoniae* alone (*n* = 144, 55 BMD-resistant) sensitivity was 45.5% (25/55) and specificity 95.5% (85/89), with four major errors concentrated in this species. Full performance metrics are summarised in Figure 4 and Table 2.

### 2.4. Skipped-Well Subgroup and Sensitivity Analysis

Eleven *K. pneumoniae* isolates (7.6% of the species cohort) showed a skipped-well pattern on BMD reading, compatible with but not diagnostic of heteroresistance. All 11 were retested in independent BMD runs and reconfirmed. Following conservative MIC assignment, every isolate in this subgroup was categorised as resistant (MIC range 4–128 mg/L). Notably, all 11 were misclassified as susceptible by VITEK 2 Compact and accounted for roughly one third of the 32 vME events observed in the species. Among non-skipped resistant *K. pneumoniae* isolates, VITEK 2 sensitivity rose to 56.8% (25/44), which remained well below the ISO 20776-2 performance benchmark.

In a pre-specified sensitivity analysis excluding the eleven skipped-well isolates (*n* = 242), the very major error rate for VITEK 2 Compact fell from 56.1% to 45.7% (21/46; 95% CI 32.1–59.7), which remains fifteen-fold above the ISO 20776-2 acceptance threshold of 3%. The direction and clinical interpretation of the result are unchanged whether or not skipped-well isolates are retained in the primary analysis.

### 2.5. Clinical Significance of the Diagnostic Discordance

The clinical importance of these diagnostic errors becomes clearer when considered against the pharmacodynamic profile of colistin. Among the 55 BMD-resistant *K. pneumoniae* isolates, 19 (34.5%) exhibited low-level resistance (MIC 4–16 mg/L), 26 (47.3%) moderate-level (32–64 mg/L), and 10 (18.2%) high-level resistance (≥128 mg/L). Thirty-six of these 55 resistant isolates (65.5%) had MIC values exceeding the achievable serum concentration range of 2–4 mg/L at standard dosing, rendering colistin monotherapy pharmacodynamically implausible. In practical terms, 32 patients in the present cohort with true colistin-resistant *K. pneumoniae* bacteremia would have received a susceptible report from the automated system, creating a realistic scenario of misdirected empirical therapy in a population already at high risk of adverse outcomes.

## 3. Discussion

For the clinician managing a patient with *K. pneumoniae* bacteremia, colistin susceptibility testing is not a routine microbiological data point. Rather, it is often the last available guide to appropriate therapy. The present study shows that the automated system in routine use in many clinical laboratories systematically misclassified more than half of truly colistin-resistant *K. pneumoniae* bloodstream isolates as susceptible, with a very major error rate of 12.6% overall and 20.8% in the *K. pneumoniae* subgroup, both far in excess of the ISO 20776-2 acceptance limit. Beyond the individual patient, these findings raise concerns for institutional antimicrobial stewardship, national empirical treatment policy, and the accuracy of regional AMR surveillance networks that shape public-health responses.

### 3.1. Rising Colistin Resistance in Turkish Bloodstream K. pneumoniae

We present these estimates as phenotypic resistance frequencies. The present dataset does not resolve the underlying molecular mechanism in each isolate, and we are cautious not to attribute the observed rise to any single pathway. The 38.2% phenotypic resistance frequency observed in *K. pneumoniae* blood isolates is considerably higher than earlier Turkish estimates. A recent systematic review and meta-analysis spanning two decades of national data reported a pooled prevalence of 1.63% (95% CI 1.51–3.12), with a clear temporal upward trend and markedly higher rates in studies that adopted EUCAST guidance with BMD [11]. The broader regional and European burden of carbapenem-resistant Enterobacterales infections has been quantified at over 33,000 attributable deaths per year in the EU/EEA, with disproportionate contribution from colistin-resistant *K. pneumoniae* and CRE [14]. Chromosomal modification of lipid A through *mgrB* inactivation remains the predominant molecular mechanism in Enterobacterales, although plasmid-mediated *mcr* genes account for a variable proportion of isolates depending on the regional context [15,16]. Our findings are consistent with the emerging upper bound of this national and regional trajectory. Dedicated molecular characterization of this cohort is a priority next step and is already in planning.

### 3.2. Colistin Resistance in E. coli

Colistin resistance in *E. coli* was uncommon in this cohort, with only two of 109 isolates classified as resistant by BMD (1.8%; 95% CI 0.5–6.4). This is in line with regional surveillance data, which typically report colistin resistance in *E. coli* below 2% in Europe, with higher values in some Mediterranean and North African settings linked to *mcr* dissemination. VITEK 2 Compact classified both resistant *E. coli* isolates as susceptible and showed 100% specificity, which gives apparent perfect categorical agreement among susceptible isolates but zero sensitivity in a very small resistant subgroup. A resistant denominator of two does not support a strong conclusion: these numbers are hypothesis-generating rather than definitive, and multicentre work with larger *E. coli* cohorts is needed before the VITEK 2 performance for colistin in this species can be considered characterised. The lesson of this subgroup is cautionary, that small resistant subsets with 100% specificity but zero sensitivity are easy to misread as reassuring.

### 3.3. Clinical Consequences for Individual Patient Care

The primary objective of this study was to benchmark the diagnostic performance of a widely deployed automated susceptibility system against the reference broth microdilution method, and to show how that performance translates into day-to-day clinical laboratory practice. The design is laboratory-based: patient-level clinical information, treatment choice, response to therapy and outcome were not collected and are not interpreted here. For this reason the Discussion that follows is framed around diagnostic accuracy and laboratory workflow, and clinical implications are offered as plausible mechanistic inferences from the diagnostic gap rather than as empirical outcome findings from the cohort itself.

The very major error rate of 56.1% under the ISO 20776-2 denominator documented in the present cohort is among the highest reported for VITEK 2 Compact colistin testing in a European or Middle-Eastern setting. Previous method-comparison studies have described vME rates ranging from 6.5% to 36%, with performance varying by isolate selection, calendar year, and reference method [8,17,18]. Turkish data from Aydemir and colleagues reported comparable diagnostic gaps for VITEK 2 in extensively drug-resistant Enterobacterales isolates from adult intensive-care units [19]. Although the 85.8% categorical agreement observed here may appear reassuring at first glance, this figure is inflated by the very large proportion of concordantly susceptible isolates; when analysis is restricted to the clinically critical resistant subgroup, sensitivity falls to 45.6%. This phenomenon, sometimes called concordance inflation, warrants explicit reporting in method-comparison studies because it materially affects clinical interpretation. The clinical weight of these errors is best understood against prior evidence that early appropriate antimicrobial therapy is a major determinant of survival in Gram-negative sepsis [20], and against the already high baseline mortality of carbapenem-resistant *K. pneumoniae* bacteremia.

### 3.4. Heteroresistance and the Skipped-Well Signal

The eleven *K. pneumoniae* isolates showing skipped-well patterns deserve individual attention. The skipped-well signal is a BMD reading phenomenon and is not available on sealed automated cards such as the VITEK 2 Compact AST-N326, where only an interpreted MIC and a categorical S/R assignment are reported. Although this pattern is only suggestive of heteroresistance and formal demonstration requires population analysis profiling, its consequences for colistin therapy are well documented: subpopulations able to grow at higher colistin concentrations may selectively expand under drug pressure and drive treatment failure [9,10]. That every skipped-well isolate in our cohort was misclassified as susceptible by VITEK 2 provides a strong operational argument for using a BMD-based reflex confirmation protocol whenever such a pattern is observed. Recent work by Sánchez-León and colleagues has documented widespread heteroresistance in OXA-48–producing *K. pneumoniae* and has shown that commercial systems consistently underdetect these isolates [21].

### 3.5. Implications for Antimicrobial Stewardship

Institutional antimicrobial stewardship programmes depend on accurate resistance reporting to formulate empirical treatment protocols, to guide de-escalation decisions, and to select appropriate combination therapy [4]. In settings where colistin resistance approaches 40% in *K. pneumoniae* bloodstream isolates but is reported at only 20% by the automated system, stewardship teams would operate on a systematically distorted picture of the local resistance landscape [19,22]. This distortion increases the probability that empirical regimens will be inadequate for a substantial subset of patients and reduces the perceived urgency of investing in new therapeutic options such as ceftazidime–avibactam or cefiderocol [4]. A pragmatic first step is explicit stewardship-level acknowledgement of the automated system’s limitations and the introduction of reflex BMD confirmation for high-risk isolates.

### 3.6. Implications for Regional AMR Surveillance Networks

Beyond the individual institution, systematic under-detection by automated systems may undermine the quality of regional and national AMR surveillance data. Surveillance programmes aligned with the WHO Global Antimicrobial Resistance and Use Surveillance System (GLASS) [12] and the European Antimicrobial Resistance Surveillance Network (EARS-Net) [13] depend on accurate laboratory reporting to guide containment strategies, empirical treatment guidelines, and antibiotic policy at the national level. If more than half of colistin-resistant *K. pneumoniae* isolates are silently reported as susceptible, the true burden of resistance is systematically underestimated at every level of surveillance, from the local hospital antibiogram to the WHO regional dashboard. The surveillance distortion we describe is an inference from a single institution and should be verified by multicentre data before being used to adjust national or regional AMR reporting. Surveillance networks operating in high-prevalence regions may wish to recommend BMD-based confirmation of colistin susceptibility for *K. pneumoniae* bloodstream isolates entered into their datasets, and should flag automated results as method-dependent estimates rather than gold-standard values.

### 3.7. Comparison with Contemporary Literature

Our findings align with a growing body of literature. Antony and colleagues reported that VITEK misclassified a substantial fraction of colistin-resistant carbapenem-resistant *K. pneumoniae* isolates in a 2024 Indian tertiary-care comparative study [22]. A 2025 systematic review and meta-analysis by Khoshnood and colleagues concluded that colistin heteroresistance is present in a substantial proportion of *K. pneumoniae* isolates and is systematically underdetected by automated methods [23]. Turkish data from Aydın Uysal and Tünger similarly reported diagnostic discrepancies between BMD and commercial systems for colistin in *K. pneumoniae* strains, in agreement with our findings [24]. The present study extends these observations to consecutive bloodstream isolates and provides additional evidence on the clinical, stewardship, and surveillance implications of the diagnostic gap in a Turkish setting.

### 3.8. Limitations

Several limitations should be acknowledged. First, the study was conducted at a single tertiary-care institution, which inevitably limits the generalisability of the resistance prevalence estimates to community hospitals and to lower-prevalence settings. Multicentre validation across diverse Turkish and Balkan institutions would strengthen the external validity of both the epidemiological and diagnostic-performance findings.

Second, a key limitation is the absence of molecular characterization. Chromosomal *mgrB* sequencing and screening for *mcr-1* through *mcr-10* were outside the scope of the present diagnostic performance study. Without molecular data we cannot distinguish, for individual isolates, between chromosomal and plasmid-mediated colistin resistance, and the epidemiological interpretation of the observed resistance frequency remains partly speculative. A follow-up study with whole-genome sequencing of the resistant subset is in planning.

Third, population analysis profiling, the gold-standard method for formal confirmation of heteroresistance, was not performed. We therefore describe the skipped-well pattern as suggestive of, rather than diagnostic for, heteroresistance. The reader should interpret the skipped-well subgroup accordingly.

Fourth, only bloodstream isolates were analysed. Whether the diagnostic gap observed for the VITEK 2 Compact system applies with the same magnitude to respiratory, urinary, or wound isolates remains an open question. A single VITEK 2 card configuration (AST-N326) was used, and the relative performance of alternative card types and of newer automated platforms could not be evaluated in the present design.

Fifth, clinical outcome data linking VITEK 2 misclassification to treatment failure or mortality were not collected in the present observational design. The patient-safety inference discussed above rests on prior evidence that early appropriate therapy is a major determinant of outcome in Gram-negative sepsis; direct outcome-linked validation requires prospective cohort studies designed for that purpose.

## 4. Materials and Methods

### 4.1. Study Design and Setting

This prospective observational study was conducted at the Department of Medical Microbiology of a tertiary-care hospital in central Anatolia, Türkiye. The study was conducted under all required administrative and ethics committee permissions obtained from the participating institutions. All consecutive non-duplicate blood culture isolates of *K. pneumoniae* and *E. coli* obtained between July 2022 and March 2023 were prospectively accessioned for routine clinical susceptibility testing under the diagnostic clinical consent in place at the time of each admission and under the hospital’s standing institutional authorisations for routine diagnostic microbiology; residual, de-identified isolates were archived at −80 °C under standard laboratory procedures and were not accessed for any purpose during storage. The research use of these archived, non-identifiable isolates (the paired BMD and VITEK 2 Compact retesting and the comparative analysis presented here) was approved in advance of the research work by the Non-Interventional Clinical Research Ethics Committee of Kayseri City Hospital on 12 June 2026 (Approval No. 350), and the paired reference-method and automated-system testing was carried out on the archived strain collection between July and September 2026. The Committee waived patient consent for the research use because only archived, non-identifiable bacterial isolates from routine diagnostic workflow were used and no patient-identifiable data were accessed. The manuscript is reported in accordance with the STROBE guideline for observational studies. Duplicate isolates from the same patient within a 14-day window were excluded to reflect distinct infection episodes.

### 4.2. Bacterial Isolate Collection and Species Identification

Blood cultures were processed using the BACT/ALERT^®^ system (bioMérieux, Marcy-l’Étoile, France). Positive bottles were subcultured on 5% sheep blood agar (Blood Agar Base; Merck, Darmstadt, Germany), MacConkey agar, and eosin–methylene blue agar (Oxoid, Basingstoke, UK), and incubated at 35 °C for 18–24 h in ambient air. All isolates were stored at −80 °C in tryptic soy broth with 15% glycerol until further testing.

### 4.3. Broth Microdilution (Reference Method)

BMD was performed in strict accordance with EUCAST guidance for colistin testing [7]. Cation-adjusted Mueller–Hinton broth (Sigma-Aldrich, St. Louis, MO, USA) was dispensed into untreated polypropylene microtitre plates (Greiner Bio-One, Kremsmünster, Austria) to prevent colistin adsorption. Twofold serial dilutions of colistin sulfate (Sigma-Aldrich, purity ≥ 95%) were prepared over the concentration range of 0.125–128 mg/L. Bacterial inocula were prepared from overnight Mueller–Hinton agar subcultures, adjusted to 0.5 McFarland turbidity, and diluted to achieve a final inoculum of 5 × 10^5^ colony-forming units per mL. Plates were incubated at 35 °C for 16–20 h in ambient air. *E. coli* ATCC 25922 (susceptible) and *E. coli* NCTC 13846 (*mcr-1*-positive, MIC 4 mg/L) served as concurrent quality-control strains for every batch, and runs were accepted only when QC MICs remained within the EUCAST QC range. Isolates displaying a skipped-well pattern (*n* = 11) were retested in independent runs, and the highest observed MIC value was assigned when the inter-well discrepancy was limited to a single twofold dilution.

### 4.4. VITEK 2 Compact (Comparator Method)

Parallel colistin susceptibility testing was conducted using the VITEK 2 Compact system (bioMérieux, Marcy-l’Étoile, France) with AST-N326 cards, following the manufacturer’s instructions. Bacterial inocula were prepared from the same overnight subcultures used for BMD, adjusted to 0.5–0.63 McFarland in 0.45% sterile saline, and processed within 30 min of preparation. Operators performing VITEK 2 testing were blinded to the corresponding BMD result. The Advanced Expert System software (version 8.01) was used with colistin reporting parameters set per EUCAST v12.0 breakpoints. 

### 4.5. Categorical Interpretation

Categorical interpretation followed EUCAST v12.0 clinical breakpoints for Enterobacterales: susceptible (S), MIC ≤ 2 mg/L; resistant (R), MIC > 2 mg/L. No intermediate category exists for colistin under EUCAST v12.0, and no “susceptible, increased exposure” assignments were made. Categorical agreement (CA) was calculated as the proportion of isolates for which VITEK 2 and BMD agreed, over the total cohort. The major error (ME) rate was computed as the proportion of BMD-susceptible isolates misclassified as resistant by VITEK 2 (ME count/BMD-susceptible denominator), and the very major error (vME) rate as the proportion of BMD-resistant isolates misclassified as susceptible by VITEK 2 (vME count/BMD-resistant denominator), in accordance with ISO 20776-2:2021 [25]. The ISO 20776-2 acceptance threshold for vME is 3%.

### 4.6. Statistical Analysis

Statistical analyses were performed using IBM SPSS Statistics version 26.0 (IBM Corp., Armonk, NY, USA) and R version 4.3.1 (R Foundation for Statistical Computing, Vienna, Austria). All proportions are reported with Wilson 95% confidence intervals (CIs). Between-species comparison of resistance prevalence used the Fisher exact test with odds ratio (OR) and 95% CI. The observed vME rate was compared with the ISO 20776-2 acceptable threshold of 3% by one-sided exact binomial test. A pre-specified sensitivity analysis recomputed the primary diagnostic metrics after exclusion of the skipped-well subgroup (see Section 3.4). Systematic categorical discordance between BMD and VITEK 2 was assessed by the exact McNemar test, and chance-corrected agreement was quantified by Cohen’s κ coefficient. Sensitivity, specificity, positive predictive value, and negative predictive value were calculated using BMD as the reference standard. All analyses were performed in Python (version 3.11) with SciPy (version 1.11) and statsmodels (version 0.14); two-sided *p* values below 0.05 were considered statistically significant.

## 5. Conclusions

In a Turkish tertiary-care cohort of 253 bloodstream isolates, phenotypic colistin resistance was identified in 38.2% of *K. pneumoniae* and 1.8% of *E. coli* by broth microdilution. The VITEK 2 Compact system, used widely for high-throughput screening, misclassified more than half of truly resistant *K. pneumoniae* isolates as susceptible, with a very major error rate above the ISO 20776-2 acceptance threshold. These findings are consistent with existing EUCAST and CLSI guidance that broth microdilution should remain the reference standard for colistin susceptibility testing. In high-prevalence settings, laboratories using automated platforms should ensure that reference-method confirmation is available for critical isolates, particularly in *K. pneumoniae* bacteremia and when a skipped-well pattern is observed. The two methods are complementary rather than competing: automated systems retain value for screening and workflow, and BMD provides the categorical anchor that clinical decision-making and AMR surveillance require.

## Figures and Tables

**Table 1:** Cohort characteristics of *K. pneumoniae* and *E. coli* bloodstream isolates and colistin resistance profile (*n* = 253).

Characteristic	*K. pneumoniae* (*n* = 144)	*E. coli* (*n* = 109)	Total (*n* = 253)
**Number of isolates, *n* (%)**	144 (56.9)	109 (43.1)	253 (100.0)
**BMD resistant, *n* (%) [95% CI]**	55 (38.2) [30.7–46.3]	2 (1.8) [0.5–6.4]	57 (22.5) [17.8–28.1]
**MIC_50_ (mg/L)**	1	0.5	—
**MIC_90_ (mg/L)**	64	1	—
**MIC range (mg/L)**	0.25 to >128	0.25 to 8	—
**Skipped-well pattern, *n* (%)**	11 (7.6)	0 (0.0)	11 (4.3)
**VITEK 2 resistant, *n* (%) [95% CI]**	29 (20.1) [14.4–27.4]	0 (0.0) [0.0–3.4]	29 (11.5) [8.1–16.0]

*BMD, broth microdilution; CI, Wilson 95% confidence interval; MIC, minimum inhibitory concentration. Resistance defined per EUCAST v12.0 breakpoints (MIC > 2 mg/L).*

**Table 2:** Diagnostic performance of VITEK 2 Compact for colistin susceptibility, using BMD as the reference method (*n* = 253).

Performance Metric	*K. pneumoniae* (*n* = 144)	*E. coli* (*n* = 109)	Total (*n* = 253)
**Categorical agreement, % [95% CI]**	76.4 [68.8–82.6]	98.2 [93.6–99.5]	85.8 [80.9–89.5]
**Very major error (vME), % [95% CI]**	54.5 [41.3–67.3]	100 (2/2)	56.1 [43.3–68.3]
**ISO 20776-2 vME threshold (≤3%)**	—	—	Not met
**Major error (ME), % [95% CI]**	2.8 [1.1–6.9]	0.0 [0.0–3.4]	1.6 [0.6–4.0]
**Cohen κ**	0.472	—	0.506
**Sensitivity, % (TP/R)**	45.5 (25/55)	0.0 (0/2)	43.9 (25/57)
**Specificity, % (TN/S)**	95.5 (85/89)	100 (107/107)	98.0 (192/196)
**Positive predictive value, %**	96.8	—	86.2
**Negative predictive value, %**	77.9	98.2	85.7
**McNemar test, *p***	—	—	1.9 × 10^−6^

*BMD, broth microdilution; CI, Wilson 95% confidence interval; ME, major error (BMD-S, VITEK-R); TN, true negative; TP, true positive; vME, very major error (BMD-R, VITEK-S). Sensitivity and specificity calculated for the resistance endpoint. The exact McNemar test was used for paired categorical comparisons.*

## Data Availability

The de-identified isolate-level dataset containing paired BMD and VITEK 2 Compact MIC results for all *n* = 253 isolates is available from the corresponding author on reasonable request.
